# Advances of gene therapy for primary immunodeficiencies

**DOI:** 10.12688/f1000research.7512.1

**Published:** 2016-03-09

**Authors:** Fabio Candotti

**Affiliations:** 1 Division of Immunology and Allergy, University Hospital of Lausanne, Lausanne, Switzerland

**Keywords:** Gene Therapy, Primary immunodeficiency diseases, Immunodeficiencies, X-linked severe combined immunodeficiency, SCID

## Abstract

In the recent past, the gene therapy field has witnessed a remarkable series of successes, many of which have involved primary immunodeficiency diseases, such as X-linked severe combined immunodeficiency, adenosine deaminase deficiency, chronic granulomatous disease, and Wiskott-Aldrich syndrome. While such progress has widened the choice of therapeutic options in some specific cases of primary immunodeficiency, much remains to be done to extend the geographical availability of such an advanced approach and to increase the number of diseases that can be targeted. At the same time, emerging technologies are stimulating intensive investigations that may lead to the application of precise genetic editing as the next form of gene therapy for these and other human genetic diseases.

## Introduction

**Figure 1.**
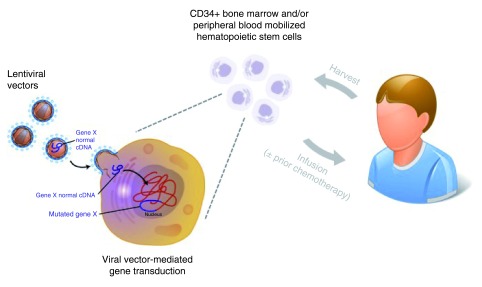
Schematic representation of a typical gene therapy procedure for primary immunodeficiency diseases (PIDs). CD43+ hematopoietic progenitors are obtained through bone marrow harvest or peripheral blood apheresis after pharmacological mobilization. Cells are then cultured *in vitro* with cytokines and growth factors (e.g. SCF, TPO, and Flt-3 ligand) and exposed to viral vectors. Finally, transduced cells are collected and reinfused to the patient through a peripheral vein. If the gene therapy protocol involves myeloreductive chemotherapy, the cytoreductive agent is administered ~24 hours before the infusion of gene-corrected cells. (Graphics modified from original illustrations by Derryl Leja, NHGRI, Image Gallery, www.genome.gov).

Primary immunodeficiency diseases (PIDs) are a heterogeneous group of mostly rare genetic diseases comprising over 250 different clinical entities and resulting from a vast variety of aberrations affecting the biological pathways of development and differentiation of the immune system ^1^. The most severe forms of PIDs are characterized by recurrent and life-threatening infections, the risk of which can be obviated only with the reconstitution of a normally functioning immune system. Since the late 1960s, allogeneic hematopoietic stem cell transplantation (HSCT) has been successfully used to treat severe PIDs and it still represents the treatment of choice. While its results have been improving steadily over the past few decades, HSCT remains an intensive procedure burdened by significant morbidity and mortality, especially when affected patients cannot benefit from HLA-identical sibling donors ^2^. Based on the notion that genetic correction of autologous hematopoietic stem cells (HSCs) could provide a safer alternative for any patient from whom HSCs can be obtained, gene therapy approaches for PIDs were developed starting in the mid-1980s and were initially based on the use of gene transfer vectors derived from murine gamma-retroviruses ^3^. These pioneer clinical protocols made their entry into the clinical arena in the early 1990s and focused on patients affected with adenosine deaminase (ADA)-deficient severe combined immunodeficiency (SCID) who derived limited benefit from the genetic correction of either their peripheral blood lymphocytes or CD34+ hematopoietic progenitors ^4– 7^. Following technical progress led to the identification of effective combinations of cytokines and growth factors (e.g. SCF, TPO, and Flt-3 ligand) that, together with culture supports such as fibronectin, resulted in major improvements in the ability to introduce genes into HSCs ^8, 9^. These improvements preluded to the first unambiguous successful clinical applications of gene therapy in patients affected with X-linked SCID (SCIDX-1), ADA-SCID, and Wiskott-Aldrich syndrome (WAS) ^10– 13^ ( Figure 1). Unfortunately, with the initial clear clinical benefits, the first serious complications of gene therapy also occurred. In a significant number of patients treated using murine gamma-retroviral vectors, insertional oncogenesis events driven by the presence of the powerful viral enhancer elements resulted in acute leukemias that, in some cases, have had fatal outcomes ^14– 16^. These serious adverse events have sparked a revision of the assessment of risks and benefits of integrating gene transfer as therapy for PIDs and prompted the development and application of new generations of viral vectors with increased safety characteristics.

This commentary will summarize the results of the current clinical trials that are making use of such newer vectors with the goal of continuing the expansion of successful applications of gene therapy for PIDs, while increasing the safety of clinical investigations.

## Improving the safety of gene therapy for primary immunodeficiencies

### X-linked severe combined immunodeficiency

This form of SCID is caused by mutations affecting the expression of the common gamma chain (γc) of the receptors for IL-2, IL-4, IL-7, IL-9, IL-15, and IL-21 ^17, 18^ and, similar to other SCID diseases, is characterized by combined impairment of T- and B-cell immunity and early susceptibility to overwhelming infections. Clinical gene therapy trials using murine gamma-retroviral vectors expressing γc were developed in the mid 1990s as an alternative therapeutic option to HSCT and, in the early 2000s, yielded the first convincing results that gene therapy could provide a cure for human genetic diseases ^10, 12^. Unfortunately, five out of the 20 SCIDX-1 patients treated in these trials developed T-cell leukemia between 2 and 5 years after gene therapy. In all cases, evidence pointed to the integration of the γc retroviral vector in the vicinity of oncogenes ( *LMO2* or *CCND2*) as the promoting factor due to the presence of a powerful enhancer element within the retroviral construct that is accepted to have caused aberrant oncogene activation and consequent leukemogenesis ^14, 15^.

Investigators in the field reacted to these adverse events by developing safer γc gene transfer vector alternatives. A gamma-retroviral vector devoid of enhancer sequences was demonstrated to be effective in the mouse model of SCIDX-1 ^19^ and then brought to the clinic in a consortium study including centers in Paris, Boston, Cincinnati, Los Angeles, and London. Recently published data show that seven out of eight evaluable patients achieved significant numbers of corrected, diverse, and functional circulating T-lymphocytes with temporal kinetics that did not differ from earlier γc gene therapy trials. In contrast to T cells, there was not significant correction of the B-cell compartment, with all patients remaining on immunoglobulin replacement therapy. Importantly, at 12–39 months post-gene therapy, no clonal expansions were detected and analysis of retroviral integration sites showed significantly less clustering near *LMO-2*, *EVI1*, or other lymphoid oncogenes compared to the earlier γc gene therapy trials ^20^. If confirmed after extended follow-up, these findings would indicate that the use of enhancer-deleted retroviral vectors can result in similar restoration of immune function for SCIDX-1 patients compared to first-generation gamma-retroviral vectors, while affording superior safety.

As another alternative to gamma-retroviral vectors for gene therapy of SCIDX-1 and other PIDs, investigators turned to gene transfer constructs based on human immunodeficiency virus type 1 (HIV-1) that are accepted as integrating vectors with lower potential to cause activation of oncogenes located near their genomic integration sites ^21^. A γc-expressing lentiviral vector based on HIV-1 has been developed ^22^ and is being used in a two-site clinical trial open at the St. Jude Children’s Research Center in Memphis, where typical SCIDX-1 patients will be enrolled, and at the National Institutes of Health, where atypical, older patients are treated. The latter arm of the trial uses non-myeloablative conditioning to improve the efficacy of engraftment of gene-corrected cells and has enrolled five patients with encouraging preliminary results of reconstitution of B-lymphocyte function in two patients at >2.5 years post-treatment ^23^. Whether or not lentiviral-mediated gene therapy for SCIDX-1 represents a safe and effective alternative will need to be established based on extended patient accrual and follow-up.

### Wiskott-Aldrich syndrome

WAS is an X-linked disorder with a spectrum of clinical presentations ranging from isolated mild thrombocytopenia to life-threatening bleeding episodes, severe eczema, recurrent infections, autoimmune disorders, and high incidence of lymphomas. Functional abnormalities affect all major lymphoid and myeloid cell populations and contribute to the heterogeneous and medically challenging clinical presentation of affected patients ^24^. HSCT can be curative for WAS, but its outcome is unsatisfactory when HLA-identical donors are not available ^2, 25^, which supported the development of gene therapy for this disease.

The first clinical gene therapy trial for WAS was carried out in Germany and used a gamma-retroviral vector to correct CD34+ cells from ten WAS patients, nine of whom showed significant increase of platelet counts and restoration of immune responses. Unfortunately, seven patients developed acute leukemia likely due to vector-mediated activation of the *LMO2*, *MDS1*, or *MN1* genes ^16^. Therefore, gamma-retroviral vector-mediated gene therapy of WAS appears to carry an unacceptably high level of risk of insertional oncogenesis. Providing an alternative to the use of murine gamma-retroviral vectors, *WAS* gene transfer constructs based on HIV-1 had also become available ^26^, which allowed for their application to two clinical trials, the initial results of which have been recently published. In the first trial, Italian investigators showed improvement of platelet counts, immune function, and clinical manifestations of the disease in three patients at ≥1 year after gene therapy. Importantly, comparison of retroviral and lentiviral vector integration sites in samples from the German and Italian studies showed lack of overrepresentation of sites targeting oncogenes in the Italian patient group, while demonstrating early enrichment of oncogenic targets in patients from the German trial ^27^. In the second trial, six out of seven patients treated in London and Paris also showed improvement of immune function and clinical manifestations 6–42 months after treatment, during which no vector-mediated clonal expansions were noted ^28^. Of note, for reasons that are not yet clear, neither trial resulted in reconstitution of normal platelet numbers, although bleeding episodes significantly reduced in number and severity, and treated patients became independent from transfusion and need for thrombopoiesis stimulator factors ^27, 28^. More recently, a trial using the same lentiviral vector used in the Italian and French sites described above has launched in Boston, MA, USA and has enrolled four patients as of December 2015 with similar results ^29^. Based on these observations, it can be concluded that lentiviral-mediated gene therapy for WAS is feasible and can result in significant benefit for treated patients. Clearly, however, long-term observation is warranted to confirm the superior safety of lentiviral gene transfer as an alternative treatment option for this disease.

### Chronic granulomatous disease

Gene therapy has long been considered an attractive alternative therapeutic option for X-linked chronic granulomatous disease (CGD), a genetic defect affecting the expression of the gp91phox molecule and characterized by impaired superoxide production in phagocytic cells with consequent susceptibility to life-threatening abscesses and/or granuloma formations in the skin, liver, lungs, or bone of affected patients ^30^. Early clinical trials were performed in the late 1990s with limited success due to low engraftment of gene-corrected hematopoietic progenitor cells and often only transitory functional correction of 0.5–1% of peripheral blood granulocytes ^31– 35^. A trial performed in Germany in 2004 using a gamma-retroviral vector expressing gp91phox under the transcriptional control of the spleen focus-forming virus long terminal repeat (LTR) appeared to have achieved superior results in two CGD patients when around 15% of neutrophils were found to be functionally corrected early after treatment. This fraction increased due to insertional activation of the *PRDM16* and *MDS1/EVI1* genes in clonal cell populations that expanded with time. Unfortunately, both patients eventually presented with myelodysplasia that was likely caused by the activation of the *EVI1* gene and that resulted in lethal complications ^36– 38^. The same clonal expansion was observed in two children with CGD treated in Switzerland with the same protocol with significant correction of functional neutrophils and eradication of fungal infections. In one of these two cases, the clonal expansion was also followed by the occurrence of myelodysplasia and both patients were rescued with allogeneic stem cell transplantation ^39, 40^.

Similar to what ensued after the cases of leukemogenesis in the SCIDX-1 and WAS trials, an enhancer element-devoid gamma-retroviral vector and a lentiviral vector expressing gp91phox have been developed for safer gene therapy approaches for CGD ^41, 42^ and multicenter clinical trials are planned in Europe and the USA to determine their efficacy. In addition to the needed improvements in safety, gene therapy approaches for CGD are confronting the as-yet-unexplained difficulty in achieving long-term engraftment of significant levels of transduced cells. The lack of a strong selective advantage of gene-corrected populations in this disease may imply that higher levels of HSC transduction and engraftment will be needed to obtain clinical benefit. In this respect, the gene therapy field is likely to borrow from the experience of HSCT in CGD to identify preparative conditioning regimens that are effective and well tolerated ^43^. Finally, with the aim of avoiding possible toxic effects of gp91phox expression in hematopoietic progenitors, the newer constructs for gene therapy of CGD carry myeloid-specific promoters and/or allow for microRNA-mediated post-transcriptional downregulation of expression in hematopoietic stem/progenitor cells ^42, 44^.

### Adenosine deaminase deficiency

This form of SCID is caused by genetic defects of ADA and presents with extreme reduction of lymphocyte numbers and impairment of immune functions that can lead to early death from infections ^45^. HSCT and enzyme replacement therapy (ERT) are available forms of treatment for this disease, but each has drawbacks that limit their efficacy ^46, 47^. As mentioned above, in the mid-1980s, ADA deficiency was identified as an ideal candidate disorder for trials of gene therapy. A series of clinical trials tested gamma-retroviral vector-mediated ADA gene transfer into patients’ peripheral blood T lymphocytes ^4, 5, 48– 51^, bone marrow, or cord blood HSCs ^6, 7, 52^ as an alternative treatment option to HSCT and ERT, but failed to result in self-standing improvements of the disease in treated patients.

The turning point was when the experimental protocols were changed to include administration of mild myeloreductive chemotherapy with busulfan (e.g. 4 mg/kg) or melphalan (140 mg/m ^2^), and the withholding of ERT, as steps aimed at increasing the initial advantage of gene-corrected HSCs. As shown initially by Aiuti and collaborators in Italy, this approach was revealed to be extremely effective in achieving immune reconstitution (increases in T-cell counts, normalization of T-cell function, and restoration of responses to vaccinations) in the majority of ten treated patients who remained off ERT in the long term ^11, 53^.

These encouraging results were confirmed in a similar gene therapy trial conducted in the UK, in which four out of six treated patients showed increases in T-cell and B-cell numbers, with normalization of *in vitro* lymphocyte responses and adequate immunoglobulin production in three subjects ^54, 55^.

Our own investigations performed at the Children’s Hospital Los Angeles, University of California Los Angeles, and the National Institutes of Health compared the immune reconstitution observed in four patients treated without prior administration of chemotherapy and while on ERT to that of six patients whose treatment strategy involved low-dose busulfan chemotherapy (75–90 mg/m ^2^) and withdrawal of ERT. The results demonstrated that the use of reduced-intensity conditioning favored engraftment of gene-modified stem cells and the generation of ADA-expressing lymphocytes and consequent immune reconstitution ^56^.

It is important to note that the immune recovery observed in ADA-SCID patients after gene therapy with gamma-retroviral vectors occurred in the absence of insertional oncogenesis events, which distinguishes the experience in this disease from the other PIDs discussed above. The reasons underlying this contrast remain unclear, but they may reflect biological differences between ADA, γc, and the WAS protein and their possible contributing roles in leukemogenesis. Regardless of the current safety record of gamma-retroviral vector-mediated gene therapy for ADA-SCID, compelling reasons existed to generate a newer, more efficient, and safer ADA vector, which was accomplished with the development of a lentiviral construct ^57^ that is being tested in the UK and USA with very encouraging preliminary results ^58^.

## Future prospects and challenges

Preclinical development is underway for several other forms of PID that would benefit from gene therapy approaches ( Table 1). Promising results have been obtained using lentiviral vectors to correct SCID due to RAG1, RAG2, and Artemis deficiencies in mouse and xenotransplant models ^59– 65^ and are expected to translate into clinical experiments in the near future. Gene therapy for PIDs such as purine nucleoside phosphorylase (PNP) deficiency, Janus kinase (JAK)-3-deficient SCID, and leukocyte adhesion deficiency type 1 (LAD-1) was considered and/or unsuccessfully carried out before technological advances established the current levels of feasibility of clinical gene transfer ^66– 68^. Better outcomes would be expected if these experiments were to be re-attempted at present times. For several other forms of PIDs, the tissue-restricted or finely regulated characteristics of expression of the causal genes represent significant challenges and will require additional technical progress. It is hoped that the expanding application of “gene editing” strategies (e.g. zinc-finger nucleases [ZFNs], transcription activator-like effector nucleases [TALENs], and clustered regularly interspaced short palindromic repeats [CRISPR]/CRISPR-associated endonuclease [Cas-9] technology) will ultimately provide the ability of performing precise genetic correction of PID-causing mutations, while respecting the physiological machineries of gene expression regulation and avoiding the problems of ectopic gene expression that are inherent in current “gene addition” approaches. Important proofs-of-concept have already been obtained using ZFN technology, including the repair of γc mutations in *in vitro* and *in vivo* xenotransplant models ^69, 70^ and the site-directed addition of the gp91phox complementary DNA (cDNA) in induced pluripotent stem cells (iPSCs) ^71^.

**Table 1.** Ongoing pre-clinical experimentations of gene therapy for primary immunodeficiency diseases.

	Challenges	Models*	Status
SCIDs			
Artemis deficiency	Ectopic expression toxicity?	KO mouse	*In vivo* gene correction ^63, 64^
CD3γ deficiency	Regulated gene expression	KO mouse	*In vitro* gene correction ^74^
JAK3-SCID	Biochemical effects of JAK3 overexpression	KO mouse	*In vitro* and *in vivo* gene correction; failed clinical attempt ^68, 75– 79^
RAG-1 deficiency	Balance efficacy/toxicity	KO mouse Xenotransplant	*In vivo* gene correction ^59, 61, 62, 65, 80^
RAG-2 deficiency	High gene expression necessary	KO mouse	*In vivo* gene correction ^60, 81^
Reticular dysgenesis	Expression in myeloid lineages	KO zebrafish iPSCs	*In vitro* and *in vivo* gene correction ^82^
Combined immunodeficiencies			
PNP deficiency	Non-immunological clinical complications	KO mouse	*In vitro* and *in vivo* gene correction ^66, 83^
ZAP70 deficiency	Restricted gene expression	KO mouse	*In vitro* and *in vivo* gene correction ^84– 88^
MHC class II deficiency	Regulated gene expression	KO mouse	*In vitro* gene correction ^89^
Antibody defects			
XLA	Restricted gene expression	KO mouse Xid mouse	*In vivo* gene correction ^90– 93^
X-HIM	Regulated gene expression	KO mouse	*In vitro* and *in vivo* gene correction ^94– 96^
Immune dysregulation syndromes			
Perforin deficiency	Restricted gene expression	KO mouse	*In vivo* gene correction ^97^
XLP	Regulated gene expression	KO mouse	*In vivo* gene correction ^98^
Innate immune defects			
LAD-1	Restricted gene expression No selective advantage	KO mouse CLAD dog	*In vitro* and *in vivo* gene correction; failed clinical attempt ^67, 99– 107^

*In addition to biological patient samples.

JAK3, Janus kinase 3; LAD-1, leukocyte adhesion deficiency type 1; PNP, purine nucleoside phosphorylase; RAG, recombination activating gene; X-HIM, X-linked hyper-IgM syndrome; XLA, X-linked agammaglobulinemia; XLP, X-linked lymphoproliferative syndrome; ZAP70, zeta-chain-associated protein kinase 70; KO, knockout; IPSCs, induced pluripotent stem cells.

While there are excellent prospects for the safer implementation of gene therapy for an increasing number of PIDs, it is difficult to ignore that clinical gene transfer remains a laborious procedure restricted to a very small number of highly specialized academic centers worldwide. For PIDs like ADA-SCID, SCIDX-1, and WAS, the current results make gene therapy a realistic therapeutic alternative that can be considered as part of the clinical management plan. Access to this therapeutic modality, however, is far from simple owing to financial and geographical considerations. As a possible solution, strategies are being developed that would allow hematopoietic progenitors to be collected at the patient’s local institution and sent to gene therapy centers where the gene transfer procedure would be performed. Cryopreserved, gene-corrected samples would then be sent back for infusion. The involvement of pharmaceutical and biotechnology companies would make these objectives easier to achieve, and it is encouraging that corporate interest in supporting clinical gene therapy trials for PIDs is increasing.

Thirty years after proof-of-principle experiments demonstrating the first corrections of genetic disease phenotypes *in vitro* ^72, 73^, gene transfer is fulfilling its promise by achieving convincing curative potential for a variety of human disorders. Since the very beginning of the field of human gene therapy, PIDs have played a major role in driving the evolution and implementation of the initial theoretical strategies of this discipline. Cutting-edge activity continues to characterize this area of gene therapy and will undoubtedly foster further applications against human diseases.

## Abbreviations

ADA: adenosine deaminase

CGD: chronic granulomatous disease

ERT: enzyme replacement therapy

Flt-3: fms-like tyrosine kinase

gc: gamma chain

HSC: hematopoietic stem cell

HSCT: hematopoietic stem cell transplantation

HIV-1: human immunodeficiency virus type 1

IL: interleukin

IPSCs: induced pluripotent stem cells

JAK: Janus kinase

LAD: leukocyte adhesion deficiency

PIDs: primary immunodeficiency diseases

RAG: recombination activating gene

SCF: stem cell factor

SCID: severe combined immunodeficiency

SCIDX-1: X-linked severe combined immunodeficiency

WAS: Wiskott-Aldrich syndrome

ZFNs: zinc-finger nucleases

## References

[1] Picard C Al-Herz W Bousfiha A: Primary Immunodeficiency Diseases: an Update on the Classification from the International Union of Immunological Societies Expert Committee for Primary Immunodeficiency 2015. *J Clin Immunol.* 2015.35(8):696–726. 10.1007/s10875-015-0201-1 26482257 PMC4659841

[2] Gennery AR Slatter MA Grandin L: Transplantation of hematopoietic stem cells and long-term survival for primary immunodeficiencies in Europe: entering a new century, do we do better? *J Allergy Clin Immunol.* 2010;126(3):602–10.e1–11. 10.1016/j.jaci.2010.06.015 20673987

[3] Miller AD: Retroviral vectors. *Curr Top Microbiol Immunol.* 1992;158:1–24. 1582242 10.1007/978-3-642-75608-5_1

[4] Blaese RM Culver KW Miller AD: T lymphocyte-directed gene therapy for ADA- SCID: initial trial results after 4 years. *Science.* 1995;270(5235):475–80. 10.1126/science.270.5235.475 7570001

[5] Bordignon C Notarangelo LD Nobili N: Gene therapy in peripheral blood lymphocytes and bone marrow for ADA- immunodeficient patients. *Science.* 1995;270(5235):470–5. 10.1126/science.270.5235.470 7570000

[6] Kohn DB Weinberg KI Nolta JA: Engraftment of gene-modified umbilical cord blood cells in neonates with adenosine deaminase deficiency. *Nat Med.* 1995;1(10):1017–23. 10.1038/nm1095-1017 7489356 PMC3013367

[7] Hoogerbrugge PM van Beusechem VW Fischer A: Bone marrow gene transfer in three patients with adenosine deaminase deficiency. *Gene Ther.* 1996;3(2):179–83. 8867866

[8] Kiem HP Andrews RG Morris J: Improved gene transfer into baboon marrow repopulating cells using recombinant human fibronectin fragment CH-296 in combination with interleukin-6, stem cell factor, FLT-3 ligand, and megakaryocyte growth and development factor. *Blood.* 1998;92(6):1878–86. 9731044

[9] Tisdale JF Hanazono Y Sellers SE: *Ex vivo* expansion of genetically marked rhesus peripheral blood progenitor cells results in diminished long-term repopulating ability. *Blood.* 1998;92(4):1131–41. 9694700

[10] Cavazzana-Calvo M Hacein-Bey S de Saint Basile G: Gene therapy of human severe combined immunodeficiency (SCID)-X1 disease. *Science.* 2000;288(5466):669–72. 10.1126/science.288.5466.669 10784449

[11] Aiuti A Slavin S Aker M: Correction of ADA-SCID by stem cell gene therapy combined with nonmyeloablative conditioning. *Science.* 2002;296(5577):2410–3. 10.1126/science.1070104 12089448

[12] Gaspar HB Parsley KL Howe S: Gene therapy of X-linked severe combined immunodeficiency by use of a pseudotyped gammaretroviral vector. *Lancet.* 2004;364(9452):2181–7. 10.1016/S0140-6736(04)17590-9 15610804

[13] Boztug K Schmidt M Schwarzer A: Stem-cell gene therapy for the Wiskott-Aldrich syndrome. *N Engl J Med.* 2010;363(20):1918–27. 10.1056/NEJMoa1003548 21067383 PMC3064520

[14] Hacein-Bey-Abina S Garrigue A Wang GP: Insertional oncogenesis in 4 patients after retrovirus-mediated gene therapy of SCID-X1. *J Clin Invest.* 2008;118(9):3132–42. 10.1172/JCI35700 18688285 PMC2496963

[15] Howe SJ Mansour MR Schwarzwaelder K: Insertional mutagenesis combined with acquired somatic mutations causes leukemogenesis following gene therapy of SCID-X1 patients. *J Clin Invest.* 2008;118(9):3143–50. 10.1172/JCI35798 18688286 PMC2496964

[16] Braun CJ Boztug K Paruzynski A: Gene therapy for Wiskott-Aldrich syndrome--long-term efficacy and genotoxicity. *Sci Transl Med.* 2014;6(227):227ra33. 10.1126/scitranslmed.3007280 24622513

[17] Noguchi M Yi H Rosenblatt HM: Interleukin-2 receptor gamma chain mutation results in X-linked severe combined immunodeficiency in humans. *Cell.* 1993;73(1):147–57. 10.1016/0092-8674(93)90167-O 8462096

[18] Puck JM Deschênes SM Porter JC: The interleukin-2 receptor gamma chain maps to Xq13.1 and is mutated in X-linked severe combined immunodeficiency, SCIDX1. *Hum Mol Genet.* 1993;2(8):1099–104. 10.1093/hmg/2.8.1099 8401490

[19] Thornhill SI Schambach A Howe SJ: Self-inactivating gammaretroviral vectors for gene therapy of X-linked severe combined immunodeficiency. *Mol Ther.* 2008;16(3):590–8. 10.1038/sj.mt.6300393 18180772 PMC6748866

[20] Hacein-Bey-Abina S Pai SY Gaspar HB: A modified γ-retrovirus vector for X-linked severe combined immunodeficiency. *N Engl J Med.* 2014;371(15):1407–17. 10.1056/NEJMoa1404588 25295500 PMC4274995

[21] Wu X Li Y Crise B: Transcription start regions in the human genome are favored targets for MLV integration. *Science.* 2003;300(5626):1749–51. 10.1126/science.1083413 12805549

[22] Zhou S Mody D DeRavin SS: A self-inactivating lentiviral vector for SCID-X1 gene therapy that does not activate LMO2 expression in human T cells. *Blood.* 2010;116(6):900–8. 10.1182/blood-2009-10-250209 20457870 PMC2924228

[23] De Ravin SS Wu X Theobald N: Lentiviral Hematopoietic Stem Cell Gene Therapy for Older Patients with X-Linked Severe Combined Immunodeficiency. *Blood.* 2015;126(23):261–261. Reference Source

[24] Bosticardo M Marangoni F Aiuti A: Recent advances in understanding the pathophysiology of Wiskott-Aldrich syndrome. *Blood.* 2009;113(25):6288–95. 10.1182/blood-2008-12-115253 19351959

[25] Moratto D Giliani S Bonfim C: Long-term outcome and lineage-specific chimerism in 194 patients with Wiskott-Aldrich syndrome treated by hematopoietic cell transplantation in the period 1980-2009: an international collaborative study. *Blood.* 2011;118(6):1675–84. 10.1182/blood-2010-11-319376 21659547 PMC3156052

[26] Dupré L Trifari S Follenzi A: Lentiviral vector-mediated gene transfer in T cells from Wiskott-Aldrich syndrome patients leads to functional correction. *Mol Ther.* 2004;10(5):903–15. 10.1016/j.ymthe.2004.08.008 15509508

[27] Aiuti A Biasco L Scaramuzza S: Lentiviral hematopoietic stem cell gene therapy in patients with Wiskott-Aldrich syndrome. *Science.* 2013;341(6148):1233151. 10.1126/science.1233151 23845947 PMC4375961

[28] Hacein-Bey Abina S Gaspar HB Blondeau J: Outcomes following gene therapy in patients with severe Wiskott-Aldrich syndrome. *JAMA.* 2015;313(15):1550–63. 10.1001/jama.2015.3253 25898053 PMC4942841

[29] Chu JI Henderson LA Armant M: Gene Therapy Using a Self-Inactivating Lentiviral Vector Improves Clinical and Laboratory Manifestations of Wiskott-Aldrich Syndrome. *Blood.* 2015;126:260–260. Reference Source

[30] Kang EM Marciano BE DeRavin S: Chronic granulomatous disease: overview and hematopoietic stem cell transplantation. *J Allergy Clin Immunol.* 2011;127(6):1319–26; quiz 1327–8. 10.1016/j.jaci.2011.03.028 21497887 PMC3133927

[31] Malech HL Sekhsaria S Whiting Theobald N: Prolonged detection of oxidase-positive neutrophils in the peripheral blood of five patients following a single cycle of gene therapy for chronic granulomatous disease. *Blood.* 1996;88((abstr. suppl. 1)):486a.

[32] Malech HL Horwitz ME Linton GF: Extended production of oxidase normal neutrophils in X-linked chronic granulomatous disease (CGD) following gene therapy with gp91(phox) transduced CD34 ^+^ cells. *Blood.* 1998;92:690A.9657772

[33] Goebel WS Dinauer MC: Gene therapy for chronic granulomatous disease. *Acta Haematol.* 2003;110(2–3):86–92. 10.1159/000072457 14583668

[34] Kang EM Choi U Theobald N: Retrovirus gene therapy for X-linked chronic granulomatous disease can achieve stable long-term correction of oxidase activity in peripheral blood neutrophils. *Blood.* 2010;115(4):783–91. 10.1182/blood-2009-05-222760 19965657 PMC2815517

[35] Grez M Reichenbach J Schwäble J: Gene therapy of chronic granulomatous disease: the engraftment dilemma. *Mol Ther.* 2011;19(1):28–35. 10.1038/mt.2010.232 21045810 PMC3017455

[36] Ott MG Schmidt M Schwarzwaelder K: Correction of X-linked chronic granulomatous disease by gene therapy, augmented by insertional activation of *MDS1-EVI1, PRDM16* or *SETBP1*. *Nat Med.* 2006;12(4):401–9. 10.1038/nm1393 16582916

[37] Stein S Ott MG Schultze-Strasser S: Genomic instability and myelodysplasia with monosomy 7 consequent to *EVI1* activation after gene therapy for chronic granulomatous disease. *Nat Med.* 2010;16(2):198–204. 10.1038/nm.2088 20098431

[38] Aiuti A Bacchetta R Seger R: Gene therapy for primary immunodeficiencies: Part 2. *Curr Opin Immunol.* 2012;24(5):585–91. 10.1016/j.coi.2012.07.012 22909900

[39] Bianchi M Hakkim A Brinkmann V: Restoration of NET formation by gene therapy in CGD controls aspergillosis. *Blood.* 2009;114(13):2619–22. 10.1182/blood-2009-05-221606 19541821 PMC2756123

[40] Siler U Paruzynski A Holtgreve-Grez H: Successful Combination of Sequential Gene Therapy and Rescue Allo-HSCT in Two Children with X-CGD - Importance of Timing. *Curr Gene Ther.* 2015;15(4):416–27. 10.2174/1566523215666150515145255 25981636

[41] Moreno-Carranza B Gentsch M Stein S: Transgene optimization significantly improves SIN vector titers, gp91 ^phox^ expression and reconstitution of superoxide production in X-CGD cells. *Gene Ther.* 2009;16(1):111–8. 10.1038/gt.2008.143 18784749

[42] Santilli G Almarza E Brendel C: Biochemical correction of X-CGD by a novel chimeric promoter regulating high levels of transgene expression in myeloid cells. *Mol Ther.* 2011;19(1):122–32. 10.1038/mt.2010.226 20978475 PMC3017453

[43] Güngör T Teira P Slatter M: Reduced-intensity conditioning and HLA-matched haemopoietic stem-cell transplantation in patients with chronic granulomatous disease: a prospective multicentre study. *Lancet.* 2014;383(9915):436–48. 10.1016/S0140-6736(13)62069-3 24161820

[44] Chiriaco M Farinelli G Capo V: Dual-regulated lentiviral vector for gene therapy of X-linked chronic granulomatosis. *Mol Ther.* 2014;22(8):1472–83. 10.1038/mt.2014.87 24869932 PMC4435596

[45] Hirschhorn R Grunebaum E Roifman C: Immunodeficiency Due to Defects of Purine Metabolism: Territorial Administration under Attack in Orleans and Washington. In: Hans D. Ochs, MD, Dr.med, C. I. Edvard Smith, PhD, Jennifer M. Puck, MD, editors. *Primary Immunodeficiency Diseases: A Molecular and Genetic Approach.*Oxford University Press,2013;188–230. 10.1093/med/9780195389838.003.0014

[46] Gaspar HB Aiuti A Porta F: How I treat ADA deficiency. *Blood.* 2009;114(17):3524–32. 10.1182/blood-2009-06-189209 19638621 PMC2766674

[47] Hassan A Booth C Brightwell A: Outcome of hematopoietic stem cell transplantation for adenosine deaminase-deficient severe combined immunodeficiency. *Blood.* 2012;120(17):3615–24; quiz 3626. 10.1182/blood-2011-12-396879 22791287

[48] Onodera M Ariga T Kawamura N: Successful peripheral T-lymphocyte-directed gene transfer for a patient with severe combined immune deficiency caused by adenosine deaminase deficiency. *Blood.* 1998;91(1):30–6. 9414266

[49] Misaki Y Ezaki I Ariga T: Gene-transferred oligoclonal T cells predominantly persist in peripheral blood from an adenosine deaminase-deficient patient during gene therapy. *Mol Ther.* 2001;3(1):24–7. 10.1006/mthe.2000.0232 11162307

[50] Aiuti A Vai S Mortellaro A: Immune reconstitution in ADA-SCID after PBL gene therapy and discontinuation of enzyme replacement. *Nat Med.* 2002;8(5):423–5. 10.1038/nm0502-423 11984564

[51] Muul LM Tuschong LM Soenen SL: Persistence and expression of the adenosine deaminase gene for 12 years and immune reaction to gene transfer components: long-term results of the first clinical gene therapy trial. *Blood.* 2003;101(7):2563–9. 10.1182/blood-2002-09-2800 12456496

[52] Otsu M: Update on a Japanese clinical trial of stem cell gene therapy for ADA-deficiency. *Human Gene Therapy.* 2010;21(10):1437–1437.

[53] Aiuti A Cattaneo F Galimberti S: Gene therapy for immunodeficiency due to adenosine deaminase deficiency. *N Engl J Med.* 2009;360(5):447–58. 10.1056/NEJMoa0805817 19179314

[54] Gaspar HB Bjorkegren E Parsley K: Successful reconstitution of immunity in ADA-SCID by stem cell gene therapy following cessation of PEG-ADA and use of mild preconditioning. *Mol Ther.* 2006;14(4):505–13. 10.1016/j.ymthe.2006.06.007 16905365

[55] Gaspar HB Cooray S Gilmour KC: Hematopoietic stem cell gene therapy for adenosine deaminase-deficient severe combined immunodeficiency leads to long-term immunological recovery and metabolic correction. *Sci Transl Med.* 2011;3(97):97ra80. 21865538 10.1126/scitranslmed.3002716

[56] Candotti F Shaw KL Muul L: Gene therapy for adenosine deaminase-deficient severe combined immune deficiency: clinical comparison of retroviral vectors and treatment plans. *Blood.* 2012;120(18):3635–46. 10.1182/blood-2012-02-400937 22968453 PMC3488882

[57] Carbonaro DA Zhang L Jin X: Preclinical demonstration of lentiviral vector-mediated correction of immunological and metabolic abnormalities in models of adenosine deaminase deficiency. *Mol Ther.* 2014;22(3):607–22. 10.1038/mt.2013.265 24256635 PMC3944341

[58] Gaspar B Buckland K Rivat C: Immunological and Metabolic Correction After Lentiviral Vector Mediated Haematopoietic Stem Cell Gene Therapy for ADA Deficiency. *Journal of Clinical Immunology.* 2014;34:S167–S168.

[59] Pike-Overzet K Rodijk M Ng YY: Correction of murine Rag1 deficiency by self-inactivating lentiviral vector-mediated gene transfer. *Leukemia.* 2011;25(9):1471–83. 10.1038/leu.2011.106 21617701

[60] van Til NP de Boer H Mashamba N: Correction of murine *Rag2* severe combined immunodeficiency by lentiviral gene therapy using a codon-optimized *RAG2* therapeutic transgene. *Mol Ther.* 2012;20(10):1968–80. 10.1038/mt.2012.110 22692499 PMC3464632

[61] van Til NP Sarwari R Visser TP: Recombination-activating gene 1 ( *Rag1*)-deficient mice with severe combined immunodeficiency treated with lentiviral gene therapy demonstrate autoimmune Omenn-like syndrome. *J Allergy Clin Immunol.* 2014;133(4):1116–23. 10.1016/j.jaci.2013.10.009 24332219

[62] Pike-Overzet K Baum C Bredius RG: Successful RAG1-SCID gene therapy depends on the level of RAG1 expression. *J Allergy Clin Immunol.* 2014;134(1):242–3. 10.1016/j.jaci.2014.04.033 25117803

[63] Mostoslavsky G Fabian AJ Rooney S: Complete correction of murine Artemis immunodeficiency by lentiviral vector-mediated gene transfer. *Proc Natl Acad Sci U S A.* 2006;103(44):16406–11. 10.1073/pnas.0608130103 17062750 PMC1637595

[64] Benjelloun F Garrigue A Demerens-de Chappedelaine C: Stable and functional lymphoid reconstitution in artemis-deficient mice following lentiviral artemis gene transfer into hematopoietic stem cells. *Mol Ther.* 2008;16(8):1490–9. 10.1038/mt.2008.118 18560421

[65] Lagresle-Peyrou C Benjelloun F Hue C: Restoration of human B-cell differentiation into NOD-SCID mice engrafted with gene-corrected CD34 ^+^ cells isolated from Artemis or RAG1-deficient patients. *Mol Ther.* 2008;16(2):396–403. 10.1038/sj.mt.6300353 18223550

[66] Nelson DM Butters KA Markert ML: Correction of proliferative responses in purine nucleoside phosphorylase (PNP)-deficient T lymphocytes by retroviral-mediated PNP gene transfer and expression. *J Immunol.* 1995;154(6):3006–14. 7876563

[67] Bauer TR Hickstein DD: Gene therapy for leukocyte adhesion deficiency. *Curr Opin Mol Ther.* 2000;2(4):383–8. 11249768

[68] Sorrentino BP Lu T Ihle J: A clinical attempt to treat JAK3-deficient SCID using retroviral-mediated gene transfer to bone marrow CD34 ^+^ cells. *Molecular Therapy.* 2003;7:S449 Reference Source

[69] Urnov FD Miller JC Lee Y: Highly efficient endogenous human gene correction using designed zinc-finger nucleases. *Nature.* 2005;435(7042):646–51. 10.1038/nature03556 15806097

[70] Genovese P Schiroli G Escobar G: Targeted genome editing in human repopulating haematopoietic stem cells. *Nature.* 2014;510(7504):235–40. 10.1038/nature13420 24870228 PMC4082311

[71] Zou J Sweeney CL Chou BK: Oxidase-deficient neutrophils from X-linked chronic granulomatous disease iPS cells: functional correction by zinc finger nuclease-mediated safe harbor targeting. *Blood.* 2011;117(21):5561–72. 10.1182/blood-2010-12-328161 21411759 PMC3110021

[72] Willis RC Jolly DJ Miller AD: Partial phenotypic correction of human Lesch-Nyhan (hypoxanthine-guanine phosphoribosyltransferase-deficient) lymphoblasts with a transmissible retroviral vector. *J Biol Chem.* 1984;259(12):7842–9. 6203897

[73] Kantoff PW Kohn DB Mitsuya H: Correction of adenosine deaminase deficiency in cultured human T and B cells by retrovirus-mediated gene transfer. *Proc Natl Acad Sci U S A.* 1986;83(17):6563–7. 10.1073/pnas.83.17.6563 3489233 PMC386544

[74] Sun JY Pacheco-Castro A Borroto A: Construction of retroviral vectors carrying human CD3 gamma cDNA and reconstitution of CD3 gamma expression and T cell receptor surface expression and function in a CD3 gamma-deficient mutant T cell line. *Hum Gene Ther.* 1997;8(9):1041–8. 10.1089/hum.1997.8.9-1041 9189762

[75] Candotti F Oakes SA Johnston JA: *In vitro* correction of JAK3-deficient severe combined immunodeficiency by retroviral-mediated gene transduction. *J Exp Med.* 1996;183(6):2687–92. 10.1084/jem.183.6.2687 8676091 PMC2192605

[76] Oakes SA Candotti F Johnston JA: Signaling via IL-2 and IL-4 in JAK3-deficient severe combined immunodeficiency lymphocytes: JAK3-dependent and independent pathways. *Immunity.* 1996;5(6):605–15. 10.1016/S1074-7613(00)80274-5 8986719

[77] Bunting KD Sangster MY Ihle JN: Restoration of lymphocyte function in Janus kinase 3-deficient mice by retroviral-mediated gene transfer. *Nat Med.* 1998;4(1):58–64. 10.1038/nm0198-058 9427607

[78] Bunting KD Flynn KJ Riberdy JM: Virus-specific immunity after gene therapy in a murine model of severe combined immunodeficiency. *Proc Natl Acad Sci U S A.* 1999;96(1):232–7. 10.1073/pnas.96.1.232 9874801 PMC15122

[79] Bunting KD Lu T Kelly PF: Self-selection by genetically modified committed lymphocyte precursors reverses the phenotype of JAK3-deficient mice without myeloablation. *Hum Gene Ther.* 2000;11(17):2353–64. 10.1089/104303400750038462 11096440

[80] Lagresle-Peyrou C Yates F Malassis-Séris M: Long-term immune reconstitution in RAG-1-deficient mice treated by retroviral gene therapy: a balance between efficiency and toxicity. *Blood.* 2006;107(1):63–72. 10.1182/blood-2005-05-2032 16174758

[81] Yates F Malassis-Séris M Stockholm D: Gene therapy of RAG-2-/- mice: sustained correction of the immunodeficiency. *Blood.* 2002;100(12):3942–9. 10.1182/blood-2002-03-0782 12393742

[82] Lagresle-Peyrou C Six EM Picard C: Human adenylate kinase 2 deficiency causes a profound hematopoietic defect associated with sensorineural deafness. *Nat Genet.* 2009;41(1):106–11. 10.1038/ng.278 19043416 PMC2612090

[83] Liao P Toro A Min W: Lentivirus gene therapy for purine nucleoside phosphorylase deficiency. *J Gene Med.* 2008;10(12):1282–93. 10.1002/jgm.1261 18924118

[84] Taylor N Bacon KB Smith S: Reconstitution of T cell receptor signaling in ZAP-70-deficient cells by retroviral transduction of the ZAP-70 gene. *J Exp Med.* 1996;184(5):2031–6. 10.1084/jem.184.5.2031 8920891 PMC2192882

[85] Steinberg M Swainson L Schwarz K: Retrovirus-mediated transduction of primary ZAP-70-deficient human T cells results in the selective growth advantage of gene-corrected cells: implications for gene therapy. *Gene Ther.* 2000;7(16):1392–400. 10.1038/sj.gt.3301249 10981666

[86] Otsu M Steinberg M Ferrand C: Reconstitution of lymphoid development and function in ZAP-70-deficient mice following gene transfer into bone marrow cells. *Blood.* 2002;100(4):1248–56. 10.1182/blood-2002-01-0247 12149205

[87] Adjali O Marodon G Steinberg M: *In vivo* correction of ZAP-70 immunodeficiency by intrathymic gene transfer. *J Clin Invest.* 2005;115(8):2287–95. 10.1172/JCI23966 16075064 PMC1180533

[88] Irla M Saade M Kissenpfennig A: ZAP-70 restoration in mice by *in vivo* thymic electroporation. *PLoS One.* 2008;3(4):e2059. 10.1371/journal.pone.0002059 18446234 PMC2323614

[89] Bradley MB Fernandez JM Ungers G: Correction of defective expression in MHC class II deficiency (bare lymphocyte syndrome) cells by retroviral transduction of CIITA. *J Immunol.* 1997;159(3):1086–95. 9233601

[90] Yu PW Tabuchi RS Kato RM: Sustained correction of B-cell development and function in a murine model of X-linked agammaglobulinemia (XLA) using retroviral-mediated gene transfer. *Blood.* 2004;104(5):1281–90. 10.1182/blood-2003-09-3044 15142874

[91] Moreau T Barlogis V Bardin F: Development of an enhanced B-specific lentiviral vector expressing BTK: a tool for gene therapy of XLA. *Gene Ther.* 2008;15(12):942–52. 10.1038/gt.2008.17 18323795

[92] Kerns HM Ryu BY Stirling BV: B cell-specific lentiviral gene therapy leads to sustained B-cell functional recovery in a murine model of X-linked agammaglobulinemia. *Blood.* 2010;115(11):2146–55. 10.1182/blood-2009-09-241869 20093406 PMC2844021

[93] Ng YY Baert MR Pike-Overzet K: Correction of B-cell development in Btk-deficient mice using lentiviral vectors with codon-optimized human BTK. *Leukemia.* 2010;24(9):1617–30. 10.1038/leu.2010.140 20574453

[94] Brown MP Topham DJ Sangster MY: Thymic lymphoproliferative disease after successful correction of CD40 ligand deficiency by gene transfer in mice. *Nat Med.* 1998;4(11):1253–60. 10.1038/3233 9809548

[95] Tahara M Pergolizzi RG Kobayashi H: Trans-splicing repair of CD40 ligand deficiency results in naturally regulated correction of a mouse model of hyper-IgM X-linked immunodeficiency. *Nat Med.* 2004;10(8):835–41. 10.1038/nm1086 15273748

[96] Romero Z Torres S Cobo M: A tissue-specific, activation-inducible, lentiviral vector regulated by human CD40L proximal promoter sequences. *Gene Ther.* 2011;18(4):364–71. 10.1038/gt.2010.144 21107438

[97] Carmo M Risma KA Arumugam P: Perforin gene transfer into hematopoietic stem cells improves immune dysregulation in murine models of perforin deficiency. *Mol Ther.* 2015;23(4):737–45. 10.1038/mt.2014.242 25523759 PMC4395774

[98] Rivat C Booth C Alonso-Ferrero M: SAP gene transfer restores cellular and humoral immune function in a murine model of X-linked lymphoproliferative disease. *Blood.* 2013;121(7):1073–6. 10.1182/blood-2012-07-445858 23223356 PMC3779401

[99] Wilson JM Ping AJ Krauss JC: Correction of CD18-deficient lymphocytes by retrovirus-mediated gene transfer. *Science.* 1990;248(4961):1413–6. 10.1126/science.1972597 1972597

[100] Bauer TR Jr Miller AD Hickstein DD: Improved transfer of the leukocyte integrin CD18 subunit into hematopoietic cell lines by using retroviral vectors having a gibbon ape leukemia virus envelope. *Blood.* 1995;86(6):2379–87. 7662985

[101] Bauer TR Schwartz BR Liles WC: Retroviral-mediated gene transfer of the leukocyte integrin CD18 into peripheral blood CD34 ^+^ cells derived from a patient with leukocyte adhesion deficiency type 1. *Blood.* 1998;91(5):1520–6. 9473215

[102] Yorifuji T Wilson RW Beaudet AL: Retroviral mediated expression of CD18 in normal and deficient human bone marrow progenitor cells. *Hum Mol Genet.* 1993;2(9):1443–8. 10.1093/hmg/2.9.1443 7902162

[103] Bauer TR Jr Hai M Tuschong LM: Correction of the disease phenotype in canine leukocyte adhesion deficiency using *ex vivo* hematopoietic stem cell gene therapy. *Blood.* 2006;108(10):3313–20. 10.1182/blood-2006-03-006908 16868255 PMC1895427

[104] Bauer TR Jr Allen JM Hai M: Successful treatment of canine leukocyte adhesion deficiency by foamy virus vectors. *Nat Med.* 2008;14(1):93–7. 10.1038/nm1695 18157138 PMC4675671

[105] Nelson EJ Tuschong LM Hunter MJ: Lentiviral vectors incorporating a human elongation factor 1alpha promoter for the treatment of canine leukocyte adhesion deficiency. *Gene Ther.* 2010;17(5):672–7. 10.1038/gt.2010.7 20164857 PMC3461956

[106] Hunter MJ Tuschong LM Fowler CJ: Gene therapy of canine leukocyte adhesion deficiency using lentiviral vectors with human CD11b and CD18 promoters driving canine CD18 expression. *Mol Ther.* 2011;19(1):113–21. 10.1038/mt.2010.203 20859258 PMC3017439

[107] Hunter MJ Zhao H Tuschong LM: Gene therapy for canine leukocyte adhesion deficiency with lentiviral vectors using the murine stem cell virus and human phosphoglycerate kinase promoters. *Hum Gene Ther.* 2011;22(6):689–96. 10.1089/hum.2010.130 21275758 PMC3107578

